# Pulvinar and total thalamus volumes are preserved following early monocular enucleation

**DOI:** 10.3389/fnins.2026.1832073

**Published:** 2026-06-09

**Authors:** Stefania S. Moro, Remy Cohan, Jennifer K. E. Steeves

**Affiliations:** Department of Psychology, Centre for Vision Research, Centre for Integrative and Applied Neuroscience, York University, Toronto, ON, Canada

**Keywords:** eye enucleation, monocular, pulvinar, thalamus, visual deprivation

## Abstract

**Background:**

Monocular enucleation, the surgical removal of one eye, occurs early in life and leads to changes in visual, auditory, and audiovisual processing in adulthood. These changes can be observed behaviorally, as well as through cortical structure and white matter connectivity of visual and auditory pathways. Subcortically, the thalamus is a critical sensory processing structure that modulates both unisensory and multisensory stimuli, which are later processed in the cortex. Previous studies have shown that following monocular enucleation, the lateral geniculate nucleus (LGN) is reduced in volume, although this reduction is less than predicted. In contrast, the medial geniculate body (MGB) is asymmetric but maintains its volume. Together, this may support the auditory and audiovisual enhancements observed following early monocular enucleation. Another key subcortical thalamic nucleus, the pulvinar, plays a broad role in human visual information processing and sensorimotor integration.

**Methods:**

The current study used structural MRI to anatomically localize and measure the total pulvinar and its subnuclei, as well as total thalamus volume, in individuals who had undergone early monocular enucleation during postnatal maturation compared to binocularly intact controls.

**Results:**

Overall, people with one eye demonstrated preserved pulvinar and total thalamus volumes compared to binocularly intact controls.

**Conclusion:**

The preserved structural volume of the pulvinar and total thalamus may support the intact lower-level auditory and audiovisual processing previously observed in individuals with one eye. The absence of pulvinar volume changes in this broad-function supporting, subcortical region builds on previous studies regarding thalamic plasticity after early monocular enucleation. These findings provide evidence that not all thalamic nuclei show measurable long-term volumetric alterations and that neural plasticity is both regionally and functionally dependent.

## 1 introduction

The pioneering studies of Wiesel and Hubel (1963) examined the effects of abnormal visual experience using animal models of visual deprivation and observed degeneration and reorganization within the visual system (see review in Daw, 2009). Their seminal research was followed by decades of rich research examining the consequences of abnormal visual experience from childhood clinical disorders of vision for the maturation of the human visual system, which continues today. An extensive literature exists on the disruptive effects of such childhood monocular developmental disorders as amblyopia (e.g., Freeman, 1975), strabismus (e.g., Hess and Howell, 1977), and congenital cataract (e.g., Maurer et al., 1983) on the maturation of numerous visual functions. For the most part, visual deprivation during postnatal visual system maturation has long-lasting disruptive effects on visual function. Monocular enucleation, the surgical removal of one eye, offers a different model and unique form of visual deprivation for quantifying the behavioral effects and underlying neural consequences of the loss of binocularity compared to amblyopia, strabismus, and monocular cataract. It differs from other, more common forms of early monocular deprivation, which exhibit unreliable and unbalanced competing afferent visual signals from the deprived eye to the developing visual system. Instead, monocular enucleation provides a model of total monocular deprivation with only one stream of visual input from the remaining eye to the visual system once the deprived (enucleated) eye is completely deafferented. If monocular enucleation occurs during infancy and individuals are assessed as adults, this allows for the observation of the long-term consequences of total deafferentation on system structure and function (see Kelly et al., 2013; Steeves et al., 2008 for reviews).

It has been well documented that the visual system changes in response to the loss of one eye, particularly when that loss occurs during early postnatal visual system maturation. Processing abilities in vision and hearing have been extensively examined in individuals who had one eye removed early in life, when the visual system is not yet mature. In some instances, processing is altered; in others, it remains intact. For example, visual spatial form ability is enhanced (Nicholas et al., 1996; Reed et al., 1997; Steeves et al., 2004) or remains intact (Cattaneo et al., 2014), but visual motion processing is reduced (see Steeves et al., 2008; Kelly et al., 2013 for reviews). In addition, some evidence of cross-sensory adaptation in response to the compromised enucleated visual system has been observed such as enhanced auditory spatial localization compared to binocular controls (Hoover et al., 2012) and less susceptibility to audiovisual illusions such as the McGurk Effect (Moro and Steeves, 2018a) and sound induced flash illusion (Moro and Steeves, 2018b) indicating more equivalent processing of auditory and visual information. Other studies have shown the intact integration of audiovisual stimuli (Moro et al., 2014) and the capture of dynamic visual stimuli by simulated auditory motion (Moro and Steeves, 2018c) despite a lack of typical patterns of visual dominance (Colavita effect: Moro and Steeves, 2012, 2013).

The underlying neural substrates of visual and auditory systems have been documented following the loss of one eye early in life. As expected, there is significant degeneration of the anterior visual system, including the optic chiasm, following early eye enucleation (Kelly et al., 2014). In order to examine the consequences of hemidecussation of the anterior visual pathway, one can examine the influence of the crossed and uncrossed projections from the retina of the enucleated eye and compare them to those of the dominant eye of the binocularly intact controls. The primate visual pathway shows hemidecussation, where retinal ganglion cell axons from each eye converge at the optic chiasm and the temporal retinal fibers project ipsilaterally while the nasal fibers project contralaterally via the optic tracts to each lateral geniculate nucleus (LGN) of the thalamus in segregated, eye-specific laminae (Garey and de Courten, 1983). At birth, this structure is relatively adult-like in volume and laminar organization and is morphologically mature by 9 months of age; however, it continues to develop physiologically (de Courten and Garey, 1982; Garey and de Courten, 1983; Blakemore and Vital-Durand, 1986).

Subcortically, the thalamus plays an important role in early sensory processing, modulating both unisensory and multisensory events that are later further processed in the cortex (Baier et al., 2006; Cappe et al., 2009; Musacchia et al., 2007; Jiang et al., 2013). The thalamus is composed of a collection of nuclei, each with specific processing functions, and is a key structure for sensory information processing and communication across brain areas (Guedj and Vuilleumier, 2020). Compared to binocularly intact controls, an overall decrease in LGN volume has been observed with early eye enucleation, shortly after birth (Kelly et al., 2014). This LGN volume loss is asymmetrical (Kelly et al., 2014), which is consistent with a subcortical structure that has layered inputs from each eye and that, following monocular enucleation, loses half of the visual system’s input signal due to deafferentation. This asymmetry indicates plasticity and reorganization of LGN cells. However, the LGN volumes are larger (less reduced) than expected, indicating preservation or recruitment of deafferented LGN cells (Kelly et al., 2014). In addition, the medial geniculate body (MGB) of the thalamus exhibits no overall volume difference compared to controls but is larger in the left hemisphere of individuals with early eye enucleation regardless of which eye was removed (Moro et al., 2015). This may be due to increased interaction with the primary auditory cortex. Early eye enucleation alters cortical white matter structure of the visual system (Wong et al., 2018), auditory and audiovisual systems in cortico-cortico and thalamo-cortico connectivity (Wong et al., 2019). Morphologically, people who have had one eye removed early in life have increased surface area and gyrification in the visual, auditory, and multisensory cortices compared to binocularly intact controls (Kelly et al., 2015). Functionally, at a cortical level, increased activation for audiovisual stimuli has been observed in individuals with early eye enucleation (Moro et al., 2020). Together, these findings provide evidence that, both cortically and subcortically, the visual and auditory systems exhibit various levels of reorganization after early-life loss of one eye during postnatal maturation.

The current study examines the volume of the pulvinar, the largest of the thalamic nuclei, its subnuclei, and thalamus volume, which has not been measured before, as these nuclei were not within the field of view in previously published studies using high-resolution neuroimaging with this patient group. In non-human primates, the pulvinar and visual cortex are densely interconnected, such that distinct visual cortical regions are projected directly to distinct pulvinar regions (Arcaro et al., 2015; Benvenuto and Rezak, 1976; Ungerleider et al., 1984; Schmahmann and Pandya, 1990; Adams et al., 2000; Gutierrez et al., 2000; Schipp, 2001). In humans, contralateral visual space and the fovea are represented by two visuotopic maps found in the ventral pulvinar (Arcaro et al., 2015). Parallelling the dorsal and ventral distinctions of visual function in the cortex (Milner and Goodale, 1993), the pulvinar displays a similar pattern in functional connectivity between its dorsal and ventral portions where the ventral pulvinar is more strongly connected with early visual and extrastriate cortical areas and the dorsal pulvinar is more strongly connected with the parietal and frontal cortical areas (Arcaro et al., 2015; Ungerleider et al., 1983). In addition to the feedforward retinal connections to the pulvinar, there are extensive feedback cortico-pulvinar connections that play an important role in human visual information processing, including visual–spatial attention, attention shifting, visual integration, and sensorimotor integration (Leh et al., 2008). The pulvinar is divided into subnuclei (anterior, inferior, lateral, and medial) and has widespread cortical connections, including parietal, occipital, temporal, cingulate, and prefrontal cortical areas (Guedj and Vuilleumier, 2020). The lateral and inferior subnuclei are functionally coupled with higher-level areas in dorsolateral prefrontal and parietal cortex, suggesting a more integrative role in visual information processing (Guedj and Vuilleumier, 2020). Specifically, the inferior subnucleus is associated with face and object perception, and memory (Yuan et al., 2017; Guedj and Vuilleumier, 2020).

Animal studies suggest that early loss of retinal input can influence subcortical visual thalamic development, although the evidence is stronger for the LGN than for the pulvinar. In non-human primates, prenatal bilateral enucleation reduces inferior pulvinar size, whereas the lateral pulvinar appears relatively preserved, suggesting subregion-specific rather than global pulvinar plasticity in the context of complete vision loss (Dehay et al., 1996). Assuming a similar plasticity profile may exist with human early postnatal unilateral enucleation, the inferior pulvinar may be more impacted than the lateral or other subregions of the pulvinar following monocular enucleation. This prediction would be consistent with our previously observed plasticity in the LGN and MGB thalamic nuclei following early postnatal monocular enucleation and the behavioral differences in visual, auditory, and audiovisual processing.

## 2 Methods

### 2.1 Participants

#### 2.1.1 Monocular enucleation group

Seven adult participants who had undergone monocular enucleation (ME) at The Hospital for Sick Children in Toronto, Canada, participated in this study (mean age = 34 years, SD = 13 years). All ME participants had undergone unilateral eye enucleation (four right eyes removed) due to retinoblastoma, a rare childhood cancer of the retina. Age at enucleation ranged from 4 to 60 months (mean age at enucleation = 23 months, SD = 18 months).

#### 2.1.2 Binocular viewing control group

Twenty binocularly intact controls with a mean age of 32 years (SD = 15 years) were tested. Nine control participants were right-eye dominant.

All participants (ME and BV) reported normal hearing and normal or corrected-to-normal acuity as assessed using an ETDRS eye chart (Precision Vision™, La Salle, IL), and wore optical correction as needed. All participants gave informed consent prior to inclusion in the study, which was approved by York University’s Office of Research Ethics.

### 2.2 Procedure

All participants underwent magnetic resonance imaging (MRI) at York University’s Sherman Health Science Research Centre with a Siemens MAGNETOM Tim Trio 3 T MRI scanner (Siemens, Erlangen, Germany) using a 32-channel high-resolution brain array coil. High-resolution whole-brain structural images were obtained with a T1 magnetization-prepared rapid gradient echo imaging sequence. The anatomical imaging had the following parameters: 192 slices; in-plane resolution 1 × 1 mm; slice thickness 1 mm; TR 1,900 ms; TE 2.5 ms; imaging matrix 256 × 256; flip angle 9°; FoV = 256 mm. Structural MRI images were collected as part of a larger functional MRI (fMRI) study (see Moro et al., 2020).

### 2.3 Data analysis

Structural MRI data were processed using FreeSurfer version 7.4.1 (Fischl, 2012). T1-weighted anatomical images were analyzed using the recon-all processing pipeline, which performs motion correction, intensity normalization, skull stripping, Talairach registration, cortical surface reconstruction, and volumetric segmentation of cortical and subcortical brain structures (Dale et al., 1999; Fischl, 2012).

To obtain volumetric estimates of thalamic nuclei, we applied the FreeSurfer thalamic nuclei segmentation module, which uses a probabilistic atlas derived from high-resolution *ex vivo* MRI and histological data to parcellate the thalamus into anatomically defined nuclei (Iglesias et al., 2018). This procedure generates subject-specific segmentation maps and volumetric estimates for individual thalamic nuclei.

**Figure 1:**
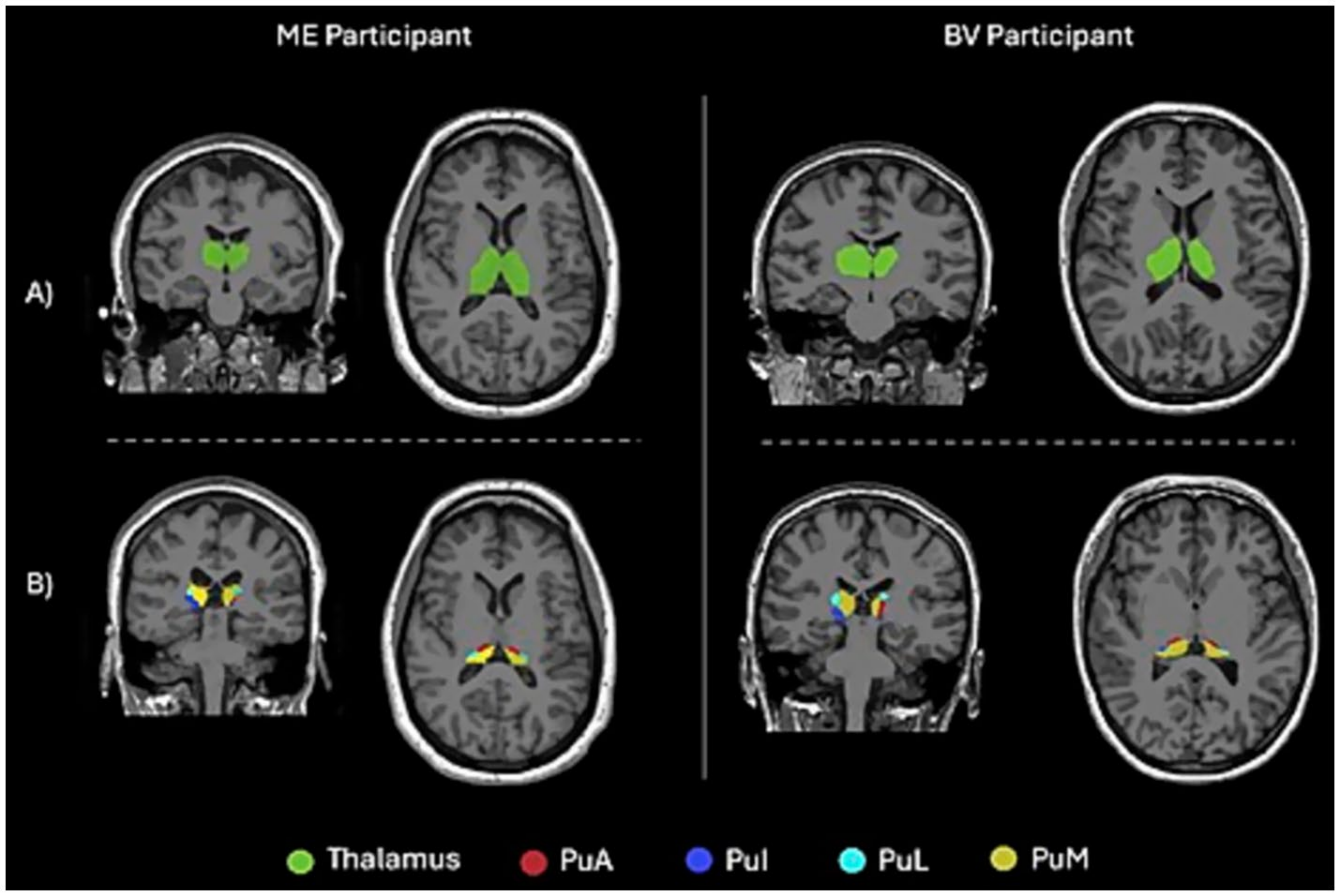
FreeSurfer segmentation of the thalamus and pulvinar in one representative ME participant (left column) and BV control participant (right column). Coronal and axial T1-weighted images are shown. **(A)** Whole-thalamic segmentation (green), including the pulvinar subdivisions. **(B)** Whole-pulvinar mask including the anterior (PuA; red), inferior (PuI; dark blue), lateral (PuL; light blue), and medial (PuM; yellow) pulvinar nuclei within each hemisphere. Images are displayed in neurological orientation.

For the purposes of the present study, analyses focused on total thalamus and pulvinar volumes. Total pulvinar volume was computed separately for each hemisphere by summing the volumes of the four pulvinar subnuclei identified by the segmentation algorithm (anterior, inferior, lateral, and medial pulvinar). Volumetric estimates were extracted from the FreeSurfer segmentation outputs and are reported in cubic millimeters (mm^3^). All segmentations were visually inspected using Freeview to visually inspect for quality control. Thalamic segmentation was examined in native anatomical space by overlaying the segmentation labels on the participant’s T1-weighted image. Representative visualizations of the thalamus and pulvinar segmentation are shown in Figure 1, illustrating examples from one ME participant and one BV control participant.

Given the hemidecussation of retinal fibers and the presence of asymmetry of subcortical structures previously found in early monocular enucleated studies (Kelly et al., 2014; Moro et al., 2015), we examined laterality of the pulvinar and thalamic structure in two ways: left vs. right hemisphere and ipsilateral vs. contralateral hemisphere to the remaining eye (ME participants) or dominant eye (BV control participants).

## 3 Results

All statistical analyses were completed using jamovi v.2.3.21 (The Jamovi Project, 2025), Prism v.9.3.1 (GraphPad Software, Inc., 2021), and R (Pinheiro et al., 2023). Analyses below focus on the pulvinar subregion, the total pulvinar volume, and the total thalamus volume. See Supplementary material for analyses of the LGN and MGB volumes. Volumetric estimates (mm^3^) were analyzed separately for each pulvinar subnucleus (anterior, inferior, lateral, medial), total pulvinar, and total thalamus and compared between participant groups (ME and BV) and hemispheres (left/ right hemisphere, as well as ipsilateral/ contralateral hemisphere to the dominant or remaining eye).

### 3.1 Left hemisphere compared to right hemisphere pulvinar subnuclei volume

#### 3.1.1 Anterior pulvinar volume

**Figure 2:**
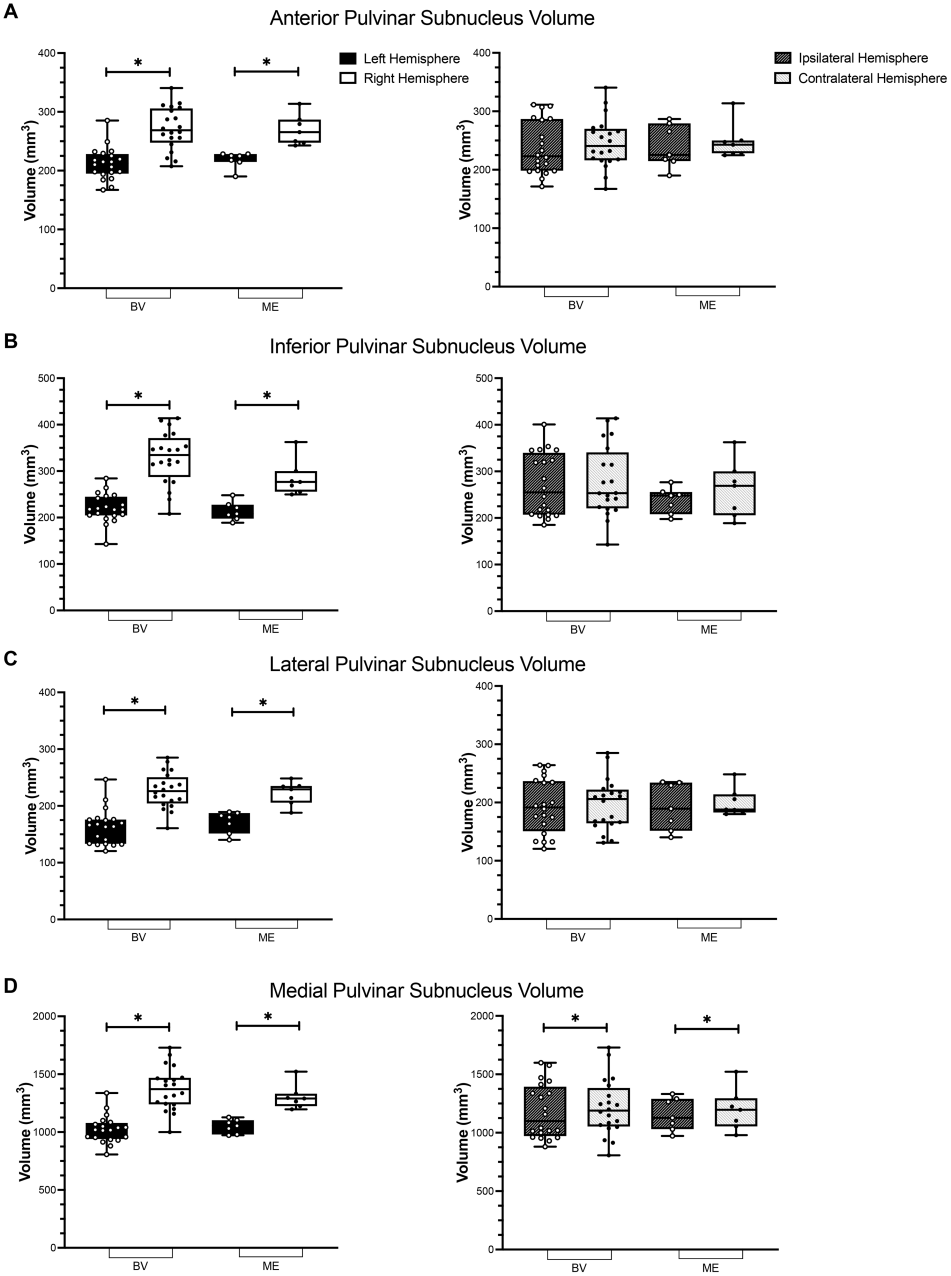
Left panel depicts volume (mm^3^) of the left (black) and right (white) hemisphere, right panel depicts volume (mm^3^) of the ipsilateral (black stripe) and contralateral (gray stripe) hemisphere to the dominant or remaining eye of the BV and ME groups. **(A)** Anterior pulvinar subnucleus, **(B)** inferior pulvinar subnucleus, **(C)** lateral pulvinar subnucleus, and **(D)** medial pulvinar subnucleus. * indicates significant difference (*p* < 0.05).

A 2 × 2 mixed model analysis of variance (ANOVA) comparing participant group (ME vs. BV) and anterior pulvinar side (left vs. right hemisphere) was conducted. There was a main effect of pulvinar side, *F*(1, 25) = 137.11, *p* < 0.001, η_p_^2^ = 0.85 and no main effect of participant group, *F*(1, 25) = 0.02, *p* = 0.89, η_p_^2^ = 0.001 or interaction, *F*(1, 25) = 1.92, *p* = 0.18, η_p_^2^ = 0.07. Bonferroni corrected post-hoc comparisons indicate that both groups have a larger right compared to left pulvinar [ME: *t*(25) = −5.99, *p* < 0.001; BV: *t*(25) = −12.86, *p* < 0.001]. These results indicate no difference in anterior pulvinar volume between participant groups. Both groups show the same laterality effect, with the right anterior pulvinar larger than the left. Figure 2A (left) plots the left and right anterior pulvinar volumes of the ME and BV groups.

#### 3.1.2 Inferior pulvinar volume

A 2 × 2 mixed model ANOVA comparing participant group (ME vs. BV) and inferior pulvinar side (left vs. right hemisphere) was conducted. There was a main effect of pulvinar side, *F*(1, 25) = 135.46, *p* < 0.001, η_p_^2^ = 0.84 and no main effect of participant group, *F*(1, 25) = 0.04, *p* = 0.85, η_p_^2^ = 0.001 or interaction, *F*(1, 25) = 0.87, *p* = 0.36, η_p_^2^ = 0.03. Bonferroni corrected post-hoc comparisons indicate that both groups have a larger right compared to left pulvinar [ME: *t*(25) = −6.22, *p* < 0.001; BV: *t*(25) = −12.35, *p* < 0.001]. These results indicate no difference in inferior pulvinar volume between participant groups. Both groups show the same laterality effect, with the right inferior pulvinar larger than the left. Figure 2B (left) plots the left and right inferior pulvinar volumes of the ME and BV groups.

#### 3.1.3 Lateral pulvinar volume

A 2 × 2 mixed model ANOVA comparing participant group (ME vs. BV) and lateral pulvinar side (left vs. right hemisphere) was conducted. There was a main effect of pulvinar side, *F*(1, 25) = 182.24, *p* < 0.001, η_p_^2^ = 0.88 and interaction, *F*(1, 25) = 4.62, *p* = 0.04, η_p_^2^ = 0.16. There was no main effect of participant group, *F*(1, 25) = 0.14, *p* = 0.72, η_p_^2^ = 0.005. Bonferroni corrected post-hoc comparisons indicate that both groups have a larger right compared to left pulvinar [ME: *t*(25) = −6.59, *p* < 0.001; BV: *t*(25) = −15.37, *p* < 0.001]. Although an interaction was observed, Bonferroni corrected post-hoc comparisons indicated that both groups had a larger right compared to left lateral pulvinar volume. These results indicate no difference in lateral pulvinar volume between participant groups. Both groups show the same laterality effect, with the right lateral pulvinar larger than the left. Figure 2C (left) plots the left and right lateral pulvinar volumes of the ME and BV groups.

#### 3.1.4 Medial pulvinar volume

A 2 × 2 mixed model ANOVA comparing participant group (ME vs. BV) and medial pulvinar side (left vs. right hemisphere) was conducted. There was a main effect of pulvinar side, *F*(1, 25) = 106.61, *p* < 0.001, η_p_^2^ = 0.81 and interaction, *F*(1, 25) = 4.67, *p* = 0.04, η_p_^2^ = 0.16. There was no main effect of participant group, *F*(1, 25) = 2.33, *p* = 0.14, η_p_^2^ = 0.09. Bonferroni corrected post-hoc comparisons indicate that both groups have a larger right compared to left pulvinar [ME: *t*(25) = −4.74, *p* < 0.001; BV: *t*(25) = −12.26, *p* < 0.001]. Although an interaction was observed, Bonferroni corrected post-hoc comparisons indicated that both groups had a larger right compared to left medial pulvinar volume. These results indicate no difference in medial pulvinar volume between participant groups. Both groups show the same laterality effect, with the right medial pulvinar larger than the left. Figure 2D (left) plots the left and right medial pulvinar volumes of the ME and BV groups.

### 3.2 Ipsilateral hemisphere compared to contralateral hemisphere pulvinar subnuclei volume

#### 3.2.1 Anterior pulvinar volume

A 2 × 2 mixed model ANOVA comparing participant group (ME vs. BV) and anterior pulvinar side (ipsilateral vs. contralateral to the remaining or dominant eye) was conducted. There was no main effect of pulvinar side, *F*(1, 25) = 0.15, *p* = 0.70, η_p_^2^ = 0.006, no main effect of participant group, *F*(1, 25) = 0.02, *p* = 0.89, η_p_^2^ = 0.001, and no interaction, *F*(1, 25) = 0.03, *p* = 0.86, η_p_^2^ = 0.001. These results indicate that there is no difference in anterior pulvinar volume between participant groups and no hemispheric difference when considering the dominant (BV group) or remaining (ME group) eye. Figure 2A (right) plots the anterior pulvinar volume ipsilateral and contralateral to the dominant or remaining eye of ME and BV groups.

#### 3.2.2 Inferior pulvinar volume

A 2×2 mixed model ANOVA comparing participant group (ME vs. BV) and inferior pulvinar side (ipsilateral vs. contralateral to the remaining or dominant eye) was conducted. There was no main effect of pulvinar side, *F*(1, 25) = 0.36, *p* = 0.55, η_p_^2^ = 0.01, no main effect of participant group, *F*(1, 25) = 0.04, *p* = 0.85, η_p_^2^ = 0.001, and no interaction, *F*(1, 25) = 0.008, *p* = 0.97, η_p_^2^ = 0.001. These results indicate that there is no difference in inferior pulvinar volume between participant groups and no hemispheric difference when considering the dominant (BV group) or remaining (ME group) eye. Figure 2B (right) plots the inferior pulvinar volume ipsilateral and contralateral to the dominant or remaining eye of ME and BV groups.

#### 3.2.3 Lateral pulvinar volume

A 2×2 mixed model ANOVA comparing participant group (ME vs. BV) and lateral pulvinar side (ipsilateral vs. contralateral to the remaining or dominant eye) was conducted. There was no main effect of pulvinar side, *F*(1, 25) = 0.22, *p* = 0.64, η_p_^2^ = 0.009, no main effect of participant group, *F*(1, 25) = 0.14, *p* = 0.72, η_p_^2^ = 0.01, and no interaction, *F*(1, 25) = 0.001, *p* = 0.97, η_p_^2^ = 0.001. These results indicate that there is no difference in lateral pulvinar volume between participant groups and no hemispheric difference when considering the dominant (BV group) or remaining (ME group) eye. Figure 2C (right) plots the lateral pulvinar volume ipsilateral and contralateral to the dominant or remaining eye of ME and BV groups.

#### 3.2.4 Medial pulvinar volume

A 2×2 mixed model ANOVA comparing participant group (ME vs. BV) and medial pulvinar side (ipsilateral vs. contralateral to the remaining or dominant eye) was conducted. There was no main effect of pulvinar side, *F*(1, 25) = 0.39, *p* = 0.54, η_p_^2^ = 0.02, no main effect of participant group, *F*(1, 25) = 2.33, *p* = 0.14, η_p_^2^ = 0.09, and no interaction, *F*(1, 25) = 0.12, *p* = 0.74, η_p_^2^ = 0.005. These results indicate that there is no difference in medial pulvinar volume between participant groups and no hemispheric difference when considering the dominant (BV group) or remaining (ME group) eye. Figure 2D (right) plots the medial pulvinar volume ipsilateral and contralateral to the dominant or remaining eye of ME and BV groups.

### 3.3 Total pulvinar volume

#### 3.3.1 Left hemisphere compared to right hemisphere total pulvinar volume

**Figure 3:**
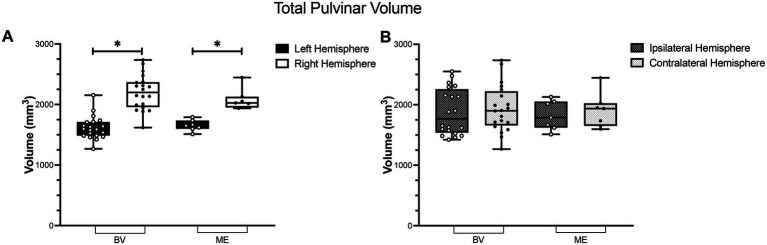
**(A)** Total pulvinar volume (mm^3^) of the left (black) and right (white) hemisphere. **(B)** Total pulvinar volume (mm^3^) of the ipsilateral (black stripe) and contralateral (gray stripe) hemisphere to the dominant or remaining eye of the BV and ME group. * indicates significant difference (*p* < 0.05).

A 2 × 2 mixed model ANOVA comparing participant group (ME vs. BV) and pulvinar side (left vs. right hemisphere) was conducted. There was a main effect of pulvinar side, *F*(1, 25) = 219.36, *p* < 0.001, η_p_^2^ = 0.89. There was no main effect of participant group, *F*(1, 25) = 0.29, *p* = 0.60, η_p_^2^ = 0.01 or interaction, *F*(1, 25) = 0.72, *p* = 0.41, η_p_^2^ = 0.03. Bonferroni corrected post-hoc comparisons indicate that both groups have a larger right compared to left pulvinar [ME: *t*(25) = −9.10, *p* < 0.001; BV: *t*(25) = −13.71, *p* < 0.001]. These results indicate no difference in total pulvinar volume between participant groups. Both groups have the same laterality effect, with a larger right than left total pulvinar. Figure 3A plots the left and right total pulvinar volumes of the ME and BV groups.

#### 3.3.2 Ipsilateral compared to contralateral total pulvinar volume

A 2 × 2 mixed model ANOVA comparing participant group (ME vs. BV) and pulvinar side (ipsilateral vs. contralateral to the remaining or dominant eye) was conducted. There was no main effect of pulvinar side, *F*(1, 25) = 0.26, *p* = 0.61, η_p_^2^ = 0.01, no main effect of participant group, *F*(1, 25) = 2.13, *p* = 0.65, η_p_^2^ = 0.008, and no interaction, *F*(1, 25) = 0.01, *p* = 0.92, η_p_^2^ = 0.001. These results indicate that there is no difference in total pulvinar volume between participant groups, nor a hemispheric difference when considering the dominant (BV group) vs. remaining (ME group) eye. Figure 3B plots the total pulvinar volume ipsilateral and contralateral to the dominant or remaining eye of ME and BV groups.

### 3.4 Total thalamus volume

#### 3.4.1 Left hemisphere compared to right hemisphere total thalamus volume

**Figure 4:**
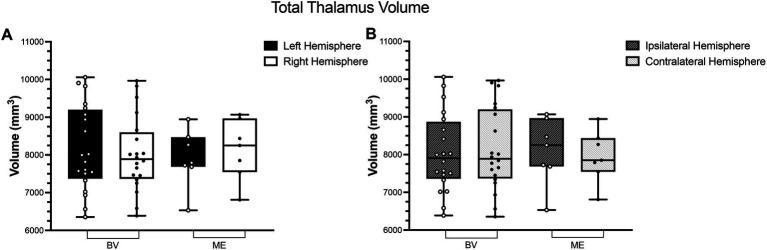
**(A)** Total thalamic volume (mm^3^) in the left (black) and right (white) hemisphere of the BV and ME groups. **(B)** Total thalamic volume (mm^3^) in the ipsilateral (black stripe) and contralateral (gray stripe) hemisphere to the dominant or remaining eye of the BV and ME groups.

A 2 × 2 mixed model ANOVA comparing participant group (ME vs. BV) and thalamus side (left vs. right hemisphere) was conducted. There was no main effect of thalamus side, *F*(1, 25) = 0.14, *p* = 0.72, η_p_^2^ = 0.01, or main effect of participant group, *F*(1, 25) = 0.02, *p* = 0.88, η_p_^2^ = 0.001. There was an interaction approaching significance, *F*(1, 25) = 4.14, *p* = 0.05, η_p_^2^ = 0.14. Bonferroni corrected post-hoc comparisons indicate no differences between participant groups or thalamus side. These results indicate that there is no difference in total pulvinar volume between participant groups, nor a hemispheric difference when not considering the dominant (BV group) or remaining (ME group) eye. Figure 4A plots the left and right thalamus volumes of the ME and BV groups.

#### 3.4.2 Ipsilateral compared to contralateral total thalamus volume

A 2 × 2 mixed model ANOVA comparing group (ME vs. BV) and thalamus side (ipsilateral vs. contralateral to the remaining or dominant eye) was conducted. There was no main effect of Thalamus Side, *F*(1, 25) = 0.09, *p* = 0.77, η_p_^2^ = 0.003; no main effect of participant group, *F*(1, 25) = 0.02, *p* = 0.88, η_p_^2^ = 0.001; or interaction, *F*(1, 25) = 1.71, *p* = 0.20, η_p_^2^ = 0.06. These results indicate that there is no difference in total thalamus volume between participant groups and no hemispheric difference when considering the dominant (BV group) or remaining (ME group) eye. Figure 4B plots the thalamus volume ipsilateral and contralateral to the dominant or remaining eye of ME and BV groups.

## 4 Discussion

The current study used structural MRI to anatomically localize and measure total and subnuclei pulvinar volumes, as well as total thalamus volume, in people with one eye who had undergone monocular enucleation early in life, compared to binocularly intact controls. Overall, people with one eye demonstrated pulvinar and thalamic volumes consistent with those of binocularly intact controls. Specifically, both controls and people with one eye exhibit the same hemispheric asymmetry in pulvinar volume, with a larger right compared to left hemisphere volume. This hemispheric asymmetry was not related to which eye was enucleated or eye dominance for binocularly intact controls. People with one eye also showed similar total thalamus volume in each hemisphere compared to binocularly intact controls, regardless of which eye was removed.

The current results indicate that despite the pulvinar’s critical subcortical role in visual processing, attention shifting, and visual integration (Leh et al., 2008) there is no alteration in structural volume in people who have had one eye surgically removed early in life compared to binocularly intact controls, despite previously observed visual and audiovisual behavioral differences (Moro and Steeves, 2012, 2013, 2018a, 2018b, 2018c, 2019; Moro et al., 2014). The lack of volume differences observed in the pulvinar and its subregions, as well as the total thalamus between these two groups, provides evidence that early eye enucleation does not alter the maturation of pulvinar and total thalamus. Although brief monocular deprivation induces short-term functional plasticity in the adult human pulvinar (Kurzawski et al., 2022), this experimental manipulation is not equivalent to the present study, which examines the long-term anatomical outcomes following permanent early monocular enucleation. These are fundamentally different forms of plasticity operating over very different timescales; accordingly, the absence of gross volumetric differences here does not preclude functional plasticity within pulvinar circuitry or related adaptive changes in other subcortical and cortical structures.

The current results differ somewhat from our previous observations of sensory system structure in people who had one eye enucleated early in life, particularly in structures that involve both auditory and visual processing. Structurally significant degeneration of the anterior visual system, including decreased optic chiasm volume and width, has been found in people with one eye compared to binocularly intact controls (Kelly et al., 2014). Subcortically, people with one eye have also shown an overall decrease in lateral geniculate nucleus (LGN) volume compared to binocularly intact controls, the LGN volume reduction was less than anticipated (Kelly et al., 2014). It would be expected that deafferentation of one eye to the LGN would reduce its volume by one-half, but this was not the case. In addition, the LGN volume contralateral to the remaining eye is even less reduced than expected (Kelly et al., 2014). People with one eye have also shown an asymmetric medial geniculate body (MGB) volume with a larger left than right MGB, regardless of which eye was enucleated, unlike binocularly intact controls (Moro et al., 2015). Despite this observed asymmetry, there was no difference in MGB volume between binocularly intact controls and people with one eye (Moro et al., 2015). Cortically, people with one eye have increased surface area and gyrification in visual, auditory, and multisensory cortices compared to binocularly intact controls (Kelly et al., 2015). Furthermore, auditory white matter tracts appear more substantial than those found in controls, and the connections between the visual and auditory systems are more intact than expected (Wong et al., 2019). Current results indicate that people who have had one eye removed early in life do not have a volumetric difference of the total thalamus in both the left and right hemispheres, regardless of which eye is removed. Despite previous studies highlighting subcortical nucleus reorganization (Kelly et al., 2014; Moro et al., 2015), our current results do not demonstrate a difference in LGN and MGB volumes compared to binocularly intact controls and further show that there is no volumetric difference in overall subcortical thalamic space. This is consistent with the previous finding of a positive relationship between MGB and LGN size, indicating that plasticity is not restricted by subcortical space confines (Moro et al., 2015). Taken together, our previously reported results and our current findings are not entirely inconsistent. Given that monocular enucleation results in complete deafferentation of a structure with layered inputs from each eye, this suggests some level of thalamic reorganization that preserves LGN volume, as mirrored in the present findings of no difference between groups. Further, both the previous and present study found no difference in MGB volume between groups, indicating preserved MGB volume with the loss of one eye. Overall, these findings may indicate reorganization within the nuclei of the thalamus that preserves their structure to a greater extent than expected. This may support intact sensory abilities in people who have had one eye removed early in life, in addition to cortical structural reorganization that involves higher-order auditory and visual processing. Together, this may perhaps contribute to this group’s intact low-level spatial and temporal audiovisual integration (Moro et al., 2014; Moro and Steeves, 2018b) and reduced susceptibility to perceiving audiovisual illusions (Moro and Steeves, 2018a; Moro and Steeves, 2018b).

Nonetheless, differences between the present findings and our earlier LGN and MGB reports should be interpreted in the context of potential patient cohort variability and a potential heterogeneous, nucleus-specific nature of thalamic reorganization following early monocular enucleation. In addition to patient cohort variability, differences in data acquisition between the present and previous studies do not permit a comparison of all thalamic nuclei across cohorts. The previous LGN (Kelly et al., 2014) and MGB volume studies (Moro et al., 2015) used high-resolution proton-density scans with a field of view (FOV) restricted to the LGN/MGB, and the pulvinar was not captured within the FOV. Future studies should continue to explore thalamic structures in this group of patients to further investigate the current patient cohort variability observed in these structures.

Given the current findings, future research aimed at investigating individual differences that relate to brain structure, function, and behavioral performance, specifically in sensory-deprived individuals, should be conducted. As is often the case when studying patients with rare diseases, it is challenging to obtain a sufficiently large sample size to allow for broader generalizations of our findings. Our study sample size was limited due to the rarity of the current patient group, and measures taken to mitigate potential limitations included sex- and age-matching each patient with a binocularly intact control participant.

In conclusion, a growing body of research demonstrates that people who have undergone monocular enucleation early in life during postnatal visual system maturation have a number of perceptual behavioral accommodations as well as structural and functional brain changes. Despite altered structural adaptations in other subcortical and cortical regions, current findings indicate preserved pulvinar and total thalamus volume in people with early monocular enucleation compared to binocularly intact controls. The lack of structural change in pulvinar volume, a broad-function supporting, subcortical region, extends previous studies on thalamic plasticity after early monocular enucleation and indicates that not all thalamic nuclei show measurable long-term volumetric alteration. This provides evidence that neural plasticity is both regionally and functionally dependent. Further investigations aimed at measuring the developmental impact of the presence of sensory accommodations may facilitate classifying the adaptive compensatory mechanisms developed to account for the loss of half of the visual input to the brain during early postnatal visual system maturation.

## Data Availability

The datasets presented in this study can be found in online repositories. The names of the repository/repositories and accession number(s) can be found at: York University Dataverse: DOI: https://doi.org/10.5683/SP3/QWMXGP.

## References

[ref1] Adams M. M. Hofpr Gattass R. Webster M. J. Ungerleider L. G. (2000). Visual cortical projections and chemoarchitecture of macaque monkey pulvinar. J. Comp. Neurol. 419, 377–393. doi: 10.1002/(SICI)1096-9861(20000410)419:3<377::AID-CNE9>3.0.CO;2-E, 10723012

[ref2] Arcaro M. J. Pinsk M. A. Kastner S. (2015). The anatomical and functional organization of the human visual pulvinar. J. Neurosci. 35, 9848–9871. doi: 10.1523/JNEUROSCI.1575-14.2015, 26156987 PMC4495241

[ref3] Baier B. Kleinschmidt A. Muller N. G. (2006). Cross-modal processing in early visual and auditory cortices depends on expected statistical relationship of multisensory information. J. Neurosci. 26, 12260–12265. doi: 10.1523/JNEUROSCI.1457-06.2006, 17122051 PMC6675430

[ref4] Benvenuto L. A. Rezak M. (1976). The cortical projections of the inferior pulvinar and adjacent lateral pulvinar in the rhesus monkey (*Macaca mulatta*): an audioradiographic study. Brain Res. 108, 1–24. doi: 10.1016/0006-8993(76)90160-8, 819095

[ref5] Blakemore C. Vital-Durand F. (1986). Effects of visual deprivation on the development of the monkey’s lateral geniculate nucleus. J. Physiol. 380, 493–511. doi: 10.1113/jphysiol.1986.sp016298, 3112372 PMC1182950

[ref6] Cappe C. Morel A. Barone P. Rouiller E. M. (2009). The thalamocortical projection systems in primate: an anatomical support for multisensory and sensorimotor interplay. Cereb. Cortex 19, 2025–2037. doi: 10.1093/cercor/bhn228, 19150924 PMC2722423

[ref7] Cattaneo Z. Bona S. Monegato M. Pece A. Vecchi T. Herbert A. M. . (2014). Visual symmetry perception in early onset monocular blindness. Vis. Cog 22, 963–974. doi: 10.1080/13506285.2014.938712

[ref8] Dale A. M. Fischl B. Sereno M. I. (1999). Cortical surface-based analysis. I. Segmentation and surface reconsruction. Neuroimage 9, 179–194. doi: 10.1006/nimg.1998.0395, 9931268

[ref9] Daw N. W. (2009). The foundations of development and deprivation in the visual system. J. Physiol. 587, 2769–2773. doi: 10.1113/jphysiol.2009.170001, 19221122 PMC2718236

[ref10] de Courten C. Garey L. J. (1982). Morphology of the neurons in the human lateral geniculate nucleus and their normal development. Exp. Brain Res. 47, 159–171. doi: 10.1007/BF00239375, 6811304

[ref11] Dehay C. Giroud P. Berland M. Killackey H. P. Kennedy H. (1996). Phenotypic characterisation of respecified visual cortex subsequent to prenatal enucleation in the monkey: development of acetylcholinesterase and cytochrome oxidase patterns. J. Comp. Neurol. 376, 386–402. doi: 10.1002/(SICI)1096-9861(19961216)376:3<386::AID-CNE3>3.0.CO;2-Z, 8956106

[ref12] Fischl B. (2012). FreeSurfer. Neuroimage 62, 774–781. doi: 10.1016/j.neuroimage.2012.01.021, 22248573 PMC3685476

[ref13] Freeman R. D. (1975). Contrast sensitivity in meridional amblyopia. Invest. Ophthal. 14, 78–81, 1110144

[ref14] Garey L. J. de Courten C. (1983). Structural development of the lateral geniculate nucleus and visual cortex in monkey and man. Beh. Brain Res. 10, 3–13. doi: 10.1016/0166-4328(83)90145-6, 6639728

[ref9001] GraphPad Software, Inc., (2021). Prism version 11.0.0 for MacOS X, Boston, Massachusetts USA: GraphPad Software. Available online at: https://www.graphpad.com.

[ref15] Guedj C. Vuilleumier P. (2020). Functional connectivity fingerprints of the human pulvinar: decoding its role in cognition. Neuroimage 221:117162. doi: 10.1016/j.neuroimage.2020.117162, 32659353

[ref16] Gutierrez C. Cola M. G. Seltzer B. Cusick C. (2000). Neurochemical and connectional organization of the dorsal pulvinar complex in monkeys. J. Comp. Neurol. 419, 61–86. doi: 10.1002/(SICI)1096-9861(20000327)419:1<61::AID-CNE4>3.0.CO;2-I, 10717640

[ref17] Hess R. F. Howell E. R. (1977). The threshold contrast sensitivity function in strabismic amblyopia: evidence for a two type classification. Vis. Res. 17, 1049–1055. doi: 10.1016/0042-6989(77)90009-8, 595414

[ref18] Hoover A. E. N. Harris L. R. Steeves J. K. E. (2012). Sensory compensation in sound localization in people with one eye. Exp. Brain Res. 216, 565–574. doi: 10.1007/s00221-011-2960-0, 22130779

[ref19] Iglesias J. E. Insausti R. Lerma-Usabiaga G. Bocchetta M. Van Leemput K. Greve D. N. . (2018). A probabilistic atlas of the human thalamic nuclei combining *ex vivo* MRI and histology. Neuroimage 183, 314–326. doi: 10.1016/j.neuroimage.2018.08.012, 30121337 PMC6215335

[ref20] Jiang F. Stecker G. C. Fine I. (2013). Functional localization of the auditory thalamus in individual human subjects. Neuroimage 78, 295–304. doi: 10.1016/j.neuroimage.2013.04.035, 23603350 PMC3672341

[ref21] Kelly K. R. DeSimone K. D. Gallie B. L. Steeves J. K. E. (2015). Increased cortical surface area and gyrification following long-term survival from early monocular enucleation. Neuroimage Clin. 7, 297–305. doi: 10.1016/j.nicl.2014.11.020, 25610793 PMC4300017

[ref22] Kelly K. R. McKetton L. Schneider K. A. Gallie B. L. Steeves J. K. E. (2014). Altered anterior visual system development following early monocular enucleation. Neuroimage Clin. 4, 72–81. doi: 10.1016/j.nicl.2013.10.014, 24319655 PMC3853349

[ref23] Kelly K. R. Moro S. S. Steeves J. K. E. (2013). “Living with one eye: plasticity in visual and auditory systems,” in Plasticity in Sensory Systems, eds. Steeves J. K. E. Harris L. R. (Cambridge: Cambridge University Press), 94–108.

[ref24] Kurzawski J. W. Lunghi C. Biagi L. Tosetti M. Morrone M. C. Binda P. (2022). Short-term plasticity in the human visual thalamus. eLife 11:e74565. doi: 10.7554/eLife.74565, 35384840 PMC9020816

[ref25] Leh S. E. Chakravarty M. M. Ptito A. (2008). The connectivity of the human pulvinar: a diffusion tensor imaging tractography study. Int. J. Biomed. Imaging 2008:789539. doi: 10.1155/2008/789539, 18274667 PMC2233985

[ref26] Maurer D. Lewis T. L. Brent H. P. (1983). Peripheral vision and optokinetic nystagmus in children with unilateral congenital cataract. Beh. Brain. Res. 10, 151–161. doi: 10.1016/0166-4328(83)90161-4, 6639723

[ref27] Milner D. A. Goodale M. A. (1993). Chapter 28: visual pathways to perception and action. Prog. Brain Res. 95, 317–337. doi: 10.1016/S0079-6123(08)60379-9, 8493342

[ref28] Moro S. S. Gorbet D. J. Steeves J. K. E. (2020). Brain activation for audiovisual information in people with one eye compared to binocular and eye-patched viewing controls. Front. Neurosci. 14:529. doi: 10.3389/fnins.2020.00529, 32508588 PMC7253581

[ref29] Moro S. S. Harris L. R. Steeves J. K. E. (2014). Optimal audiovisual processing in people with one eye. Multisens. Res. 27, 173–188. doi: 10.1163/22134808-00002453 25577901

[ref30] Moro S. S. Kelly K. R. McKetton L. Gallie B. L. Steeves J. K. E. (2015). Evidence of multisensory plasticity: asymmetrical medial geniculate body in people with one eye. Neuroimage Clin. 9, 513–518. doi: 10.1016/j.nicl.2015.09.016, 26594632 PMC4610958

[ref31] Moro S. S. Steeves J. K. E. (2012). No Colavita effect: equal auditory and visual processing in people with one eye. Exp. Brain Res. 216, 367–373. doi: 10.1007/s00221-011-2940-4, 22105335

[ref32] Moro S. S. Steeves J. K. E. (2013). No Colavita effect: increasing temporal load maintains equal auditory and visual processing in people with one eye. Neurosci. Lett. 556, 186–190. doi: 10.1016/j.neulet.2013.09.064, 24103371

[ref33] Moro S. S. Steeves J. K. E. (2018a). Audiovisual plasticity following early abnormal visual experience: reduced McGurk effect in people with one eye. Neurosci. Lett. 672, 103–107. doi: 10.1016/j.neulet.2018.02.031, 29474874

[ref34] Moro S. S. Steeves J. K. E. (2018b). Normal temporal binding window but no sound-induced flash illusion in people with one eye. Exp. Brain Res. 236, 1825–1834. doi: 10.1007/s00221-018-5263-x, 29675714

[ref35] Moro S. S. Steeves J. K. E. (2018c). Intact dynamic visual capture in people with one eye. Multisens. Res. 31, 675–688. doi: 10.1163/22134808-20181311, 31264607

[ref36] Moro S. S. Steeves J. K. E. (2019). Short and long-term visual deprivation leads to adapted use of audiovisual information for face-voice recognition. Vis. Res. 157, 274–281. doi: 10.1016/j.visres.2018.01.009, 29567099

[ref37] Musacchia G. Sams M. Skoe E. Kraus N. (2007). Musicians have enhanced subcortical auditory and audiovisual processing of speech and music. Proc. Nat. Acad. Sci. U.S.A. 104, 15894–15898. doi: 10.1073/pnas.0701498104, 17898180 PMC2000431

[ref38] Nicholas J. Heywood C. A. Cowey A. (1996). Contrast sensitivity in one-eyed subjects. Vis. Res. 36, 175–180. doi: 10.1016/0042-6989(95)00119-K, 8746251

[ref39] Pinheiro J. Bates D. DebRoy S. Sarkar D. Heisterkamp S. Van Willigen B. . (2023). Nlme: linear and nonlinear mixed effects models (version 3.1-164) [computer software]. Available online at: https://cran.r-project.org/web/packages/nlme/index.html.

[ref40] Reed M. J. Steeves J. K. E. Steinbach M. J. (1997). A comparison of contrast letter thresholds in unilateral eye enucleated subjects and binocular and monocular control subjects. Vis. Res. 37, 2465–2469. doi: 10.1016/S0042-6989(97)00034-5, 9381681

[ref41] Schipp S. (2001). Corticopulvinar connections of areas V5, V4, and V3 in the macaque monkey: a dual model of retinal and cortical topographies. J. Comp. Neurol. 439, 469–490. doi: 10.1002/cne.1363, 11596067

[ref42] Schmahmann J. D. Pandya D. N. (1990). Anatomical investigation of projections from thalamus to posterior parietal cortex in the rhesus monkey: a WGA-HRP and fluorescent tracer study. J. Comp. Neurol. 295, 299–326. doi: 10.1002/cne.902950212, 1694186

[ref43] Steeves J. K. E. González E. G. Steinbach M. J. (2008). Vision with one eye: a review of visual function following monocular enucleation. Spat. Vis. 21, 509–529. doi: 10.1163/156856808786451426, 19017480

[ref44] Steeves J. K. E. Wilkinson F. González E. G. Wilson H. R. Steinbach M. J. (2004). Global shape discrimination at reduced contrast in enucleated observers. Vis. Res. 44, 943–949. doi: 10.1016/j.visres.2003.11.015, 14992838

[ref45] The Jamovi Project (2025). Jamovi (version 2.6) [computer software]. Available online at: https://www.jamovi.org.

[ref46] Ungerleider L. G. Desimone R. Galkin T. W. Mishkin M. (1984). Subcortical projections of area MT in the macaque. J. Comp. Neurol. 223, 368–386. doi: 10.1002/cne.902230304, 6323553

[ref47] Ungerleider L. Galkin T. W. Mishkin M. (1983). Visuotopic organization of projections from striate cortex to inferior and lateral pulvinar in rhesus monkey. J. Comp. Neurol. 217, 137–157. doi: 10.1002/cne.902170203, 6886048

[ref48] Wiesel T. N. Hubel D. H. (1963). Single cell responses in striate cortex of kittens deprived of vision in one eye. J. Neurophys. 26, 1003–1017. doi: 10.1152/jn.1963.26.6.1003, 14084161

[ref49] Wong N. A. Kelly K. R. Gallie B. L. Moro S. S. Rafique S. A. Steeves J. K. E. (2018). Altered white matter structure in the visual system following earlier monocular enucleation. Hum. Brain. Map. 39, 133–144. doi: 10.1002/hbm.23831, 28963811 PMC6866261

[ref50] Wong N. A. Rafique S. A. Moro S. S. Kelly K. R. Steeves J. K. E. (2019). Altered white matter structure in auditory tracts following early monocular enucleation. Neuroimage Clin. 24:102006. doi: 10.1016/j.nicl.2019.102006, 31622842 PMC6812283

[ref51] Yuan R. Taylor P. A. Alvarez T. L. Misra D. Biswal B. B. (2017). MAPBOT: meta-analytic parcellation based on text, and its application to the human thalamus. Neuroimage 157, 716–732. doi: 10.1016/j.neuroimage.2017.06.032, 28629976 PMC6474778

