# Supplementary material for: Pulvinar and total thalamus volumes are preserved following early monocular enucleation
Source: Front Neurosci. 2026 Jun 9;20:1832073. doi: 10.3389/fnins.2026.1832073 (PMC13286922; doi:10.3389/fnins.2026.1832073)
Supplement: Supplementary file 1 [file Data_sheet_1.pdf]

Pulvinar and total thalamus volume unchanged following early monocular enucleation

Stefania S. Moro[1], Remy Cohan[1*], Jennifer K. E. Steeves[1*]

[1]Centre for Vision Research, Centre for Integrative and Applied Neuroscience, Department of Psychology, York University, Toronto, Ontario, Canada

Supplementary Material

S1.1. Lateral geniculate nucleus (LGN) volume

S1.1.1. Left hemisphere compared to right hemisphere LGN volume

A 2×2 mixed model analysis of variance (ANOVA) comparing Participant Group (ME vs BV) and LGN Side (left vs right hemisphere) was conducted. There was a main effect of LGN Side, $F(1,25) = 12.64$, $p = 0.002$, $\eta_p^2 = 0.34$ and no main effect of Participant Group, $F(1,25) = 0.21$, $p = 0.66$, $\eta_p^2 = 0.008$ or interaction, $F(1,25) = 0.07$, $p = 0.79$, $\eta_p^2 = 0.003$. Bonferroni corrected post-hoc comparisons indicate that only the BV group has a larger right compared to left LGN ($t(25) = -3.76$, $p < 0.006$). Figure S1A (left) plots left and right LGN volume of the ME and BV groups.

S1.1.2. Ipsilateral hemisphere compared to contralateral hemisphere LGN volume

A 2×2 mixed model ANOVA comparing Participant Group (ME vs BV) and LGN Side (ipsilateral vs contralateral to remaining or dominant eye) was conducted. There was no main effect of LGN Side, $F(1,25) = 0.001$, $p = 0.97$, $\eta_p^2 = 0.00$, no main effect of Participant Group, $F(1,25) = 0.21$, $p = 0.66$, $\eta_p^2 = 0.008$, and no interaction, $F(1,25) = 0.25$, $p = 0.62$, $\eta_p^2 = 0.01$. Figure S1B (right) plots the LGN volume ipsilateral and contralateral to the dominant or remaining eye of ME and BV groups.

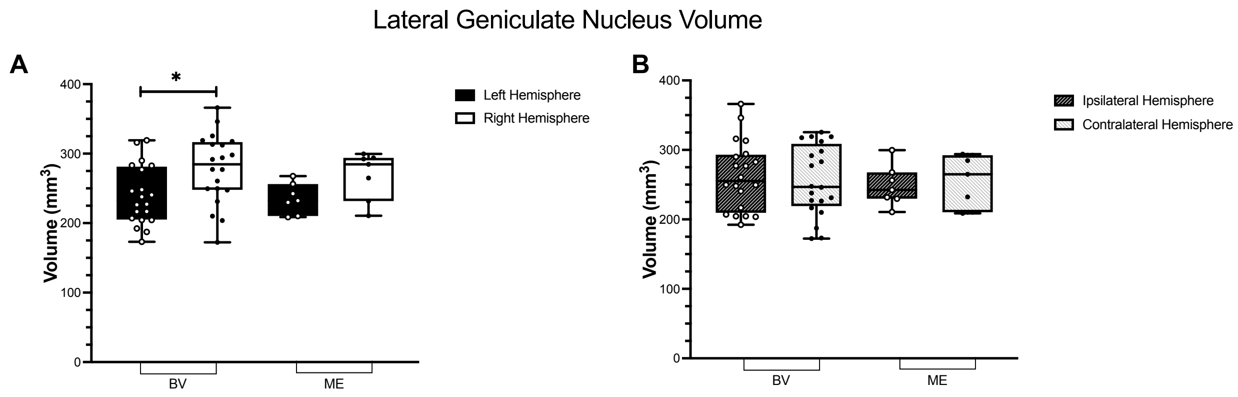

Figure S1. A. LGN volume (mm^3) of the left (black) and right (white) hemisphere; **B.** LGN volume (mm^3) of the ipsilateral (black stripe) and contralateral (grey stripe) hemisphere to the dominant or remaining eye of the BV and ME group.

S1.2. Medial geniculate body (MGB) volume

S1.2.1. Left hemisphere compared to right hemisphere MGB volume

A 2×2 mixed model ANOVA comparing Participant Group (ME vs BV) and MGB Side (left vs right hemisphere) was conducted. There was a main effect of MGB Side, $F(1,25) = 5.00$, $p = 0.035$, $\eta_p^2 = 0.17$ and no main effect of Participant Group, $F(1,25) = 0.79$, $p = 0.38$, $\eta_p^2 = 0.03$ or interaction, $F(1,25) = 2.62$, $p = 0.12$, $\eta_p^2 = 0.09$. Figure S2A (left) plots left and right MGB volume of the ME and BV groups.

S1.2.2. Ipsilateral hemisphere compared to contralateral hemisphere MGB volume

A 2×2 mixed model ANOVA comparing Participant Group (ME vs BV) and MGB Side (ipsilateral vs contralateral to remaining or dominant eye) was conducted. There was no main effect of MGB Side, $F(1,25) = 1.57$, $p = 0.22$, $\eta_p^2 = 0.06$, no main effect of Participant Group, $F(1,25) = 0.79$, $p = 0.38$, $\eta_p^2 = 0.03$, and no interaction, $F(1,25) = 0.09$, $p = 0.77$, $\eta_p^2 = 0.004$.

Figure S2B (right) plots the MGB volume ipsilateral and contralateral to the dominant or remaining eye of ME and BV groups.

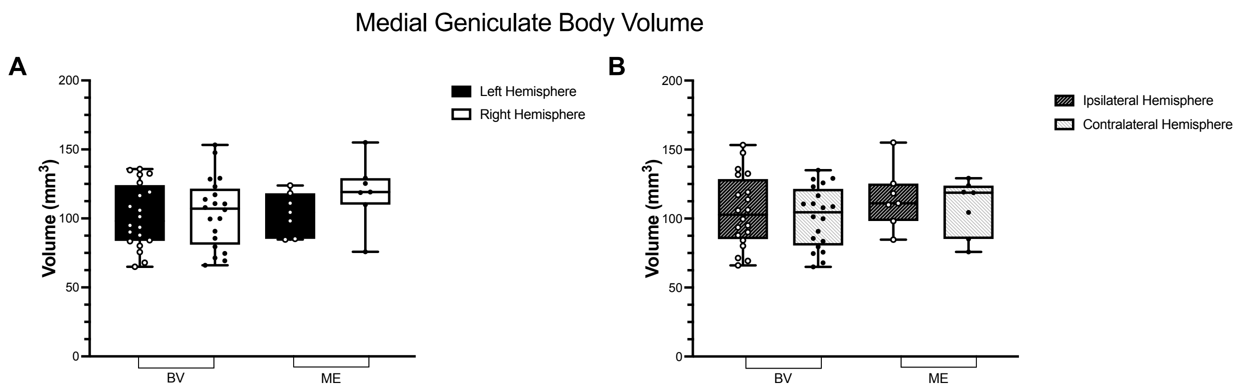

Figure S2. A. MGN volume (mm^3) of the left (black) and right (white) hemisphere; **B.** MGN volume (mm^3) of the ipsilateral (black stripe) and contralateral (grey stripe) hemisphere to the dominant or remaining eye of the BV and ME group.
